# Supplementary material for: Patterns of Intron Gain and Loss in Fungi
Source: PLoS Biol. 2004 Nov 30;2(12):e422. doi: 10.1371/journal.pbio.0020422 (PMC532390; doi:10.1371/journal.pbio.0020422)
Supplement: Table S1 — Also available at http://genes.mit.edu/NielsenEtAl/. (4.3 MB ZIP). [file pbio.0020422.st001.zip › NielsenEtAl/html/433.html]

AN2909.1.NCU06717.1.MG09535.1.FG09891.1


```
 CLUSTAL W (1.82) Multiple Sequence Alignments - Introns Inserted


Sequence 1: NCU06717.1	339 aa
Sequence 2: MG09535.1	343 aa
Sequence 3: FG09891.1	341 aa
Sequence 4: AN2909.1	340 aa
Alignment Length: 346 aa
Number Identitical Residues: 246 aa
Alignment Score (without introns) 10242


MG09535.1 	MSTALIDVDS--MEPSLQSILDQKSLRWIFVGGKGGVGKTTTSCSLAIQLAKVRRSVLLI
NCU06717.1	MSTAVINADDDHMEPTLQSILDQRSLRWIFVGGKGGVGKTTTSCSLAIQLAKVRRSVLLI
FG09891.1 	MSTALISTED-ALEPSLQSLLDQRSLRWIFVGGKGGVGKTTTSCSLAIQLAKVRRSVLLI
AN2909.1  	MSSTAIVHDDDLMEPTLQSIVNQKTLRWIFVGGKGGVGKTTTSCSLAIQLAKARKSVLLI
          	**:: *  :.. :**:***:::*::***************************.*:*****

MG09535.1 	STDPAHNLSDAFSQKFGKEARLIDGFTNLSAMEIDPNGSMQDLLAGQGADEGGAAADGMA
NCU06717.1	STDPAHNLSDAFSQKFGKEARLIDGFDNLSAMEIDPNGSIQDLLAGQGENEG---AGDMG
FG09891.1 	STDPAHNLSDAFSQKFGKEARLVNGFDNLSAMEIDPNGSIQDMLAGQGEADDVN-AAAGG
AN2909.1  	STDPAHNLSDAFGQKFGKEARLVDGFTNLSAMEVDPNGSLQDLLAN-GEGQGDDPMAGLG
          	************.*********::** ******:*****:**:**. *  :.  .    .

MG09535.1 	GMGGMMQDLAFA0IPGIDEAMSFAEVLKQVKSLSYETIIFDTAPTGHTLRFLQFPSVLEK
NCU06717.1	GVGGMMQDLAFA0IPGIDEAMSFAEVLKQVKSLSYETIIFDTAPTGHTLRFLQFPSVLEK
FG09891.1 	PLGGMMQDLAFA0IPGIDEAMSFAEVLKQVKSLSYETIVFDTAPTGHTLRFLQFPTVLEK
AN2909.1  	-VGNMMQDLAFS0IPGVDEAMSFAEVLKQVKSLSYEVIVFDTAPTGHTLRFLQFPTVLEK
          	 :*.*******: ***:*******************.*:****************:****

MG09535.1 	ALAKVSQLSSQYGPLLNGFLGGQGQLPNGQSLPEMMEKLEQLRETISEVNTQFKDENLTT
NCU06717.1	ALAKVSQLSSQYGPLLNGFLGSNGTLPNGQNLNEMMEKLETLRATISEVNTQFKDERLTT
FG09891.1 	ALAKVSQLSSQYGPLLNGFLGSGGQLPNGQNLNDMIAKLESLRETISEVNTQFQDAELTT
AN2909.1  	ALAKLSQLSSQFGPMLNSILGARGGLPGGQNLDELLQKMESLRETIGEVNTQFKNPDMTT
          	****:******:**:**.:**. * **.**.* ::: *:* ** **.******::  :**

MG09535.1 	FVCVCIAEFLSLYETERMIQELASYNIDTHCIVVNQLLFPKKGSKCDHCDARRRMQKKYL
NCU06717.1	FVCVCIPEFLSLYETERMIQELASYGIDTHSIVVNQLLFPKPGSDCEQCTARRKMQKKYL
FG09891.1 	FVCVCIAEFLSLYETERMIQELAGYGIDTHSIVVNQLLFPKKASDCDQCNARRKMQRKYL
AN2909.1  	FVCVCIAEFLSLYETERMIQELTSYQIDTHAIVVNQLLFPKQGSDCEQCNARRKMQKKYL
          	******.***************:.* ****.********** .*.*::* ***:**:***

MG09535.1 	DQIEELY-DEFNVVKMPMLIEEVRGKEKLEK~FSEMLVKPYVPPEADE
NCU06717.1	DQIEELY-DEFNVVKMPLLVEEVRGKEKLEK~FSEMLVKPFVPPQ---
FG09891.1 	DQYEELYAEDFNVVKMPLLVEEVRGKEKLEK~FSEMLVAPYVPPE---
AN2909.1  	EQIEELY-EDFNVVRMPLLVEEVRGKEKLEK2FSDMLINPYVPPN---
          	:* **** ::****:**:*:*********** **:**: *:***:
```
